# Rare copy number variant analysis in case–control studies using snp array data: a scalable and automated data analysis pipeline

**DOI:** 10.1186/s12859-024-05979-0

**Published:** 2024-11-15

**Authors:** Haydee Artaza, Ksenia Lavrichenko, Anette S. B. Wolff, Ellen C. Røyrvik, Marc Vaudel, Stefan Johansson

**Affiliations:** 1 Department of Clinical Science, University of Bergen, Bergen, Norway; 2 K.G. Jebsen Center for Autoimmune Diseases, University of Bergen, Bergen, Norway; 3 Department of Medical Genetics, Oslo University Hospital, Oslo, Norway; 4 Department of Medicine, Haukeland University Hospital, Bergen, Norway; 5 Department of Genetics and Bioinformatics, Norwegian Institute of Public Health, Bergen, Norway; 6 Mohn Center for Diabetes Precision Medicine, Department of Clinical Science, University of Bergen, Bergen, Norway; 7 Department of Pediatrics, Haukeland University Hospital, Bergen, Norway

**Keywords:** Copy number variant (CNV), Calls detection, Quality control, Burden analysis, Enrichment analysis, Rare variants analysis, Snakemake

## Abstract

**Background:**

Rare copy number variants (CNVs) significantly influence the human genome and may contribute to disease susceptibility. High-throughput SNP genotyping platforms provide data that can be used for CNV detection, but it requires the complex pipelining of bioinformatic tools. Here, we propose a flexible bioinformatic pipeline for rare CNV analysis from human SNP array data.

**Results:**

The pipeline consists of two major sub-pipelines: (1) Calling and quality control (QC) analysis, and (2) Rare CNV analysis. It is implemented in Snakemake following a rule-based structure that enables automation and scalability while maintaining flexibility.

**Conclusions:**

Our pipeline automates the detection and analysis of rare CNVs. It implements a rigorous CNV quality control, assesses the frequencies of these rare CNVs in patients versus controls, and evaluates the impact of CNVs on specific genes or pathways. We hence aim to provide an efficient yet flexible bioinformatic framework to investigate rare CNVs in biomedical research.

**Supplementary Information:**

The online version contains supplementary material available at 10.1186/s12859-024-05979-0.

## Background

Copy number variation (CNV), defined here as deletions and duplications of chromosomal segments larger than 1 kb, are a major source of genetic variation between individuals and are an essential factor in many complex diseases, including mental illness, developmental disorders, and cancer [1]. In particular, distinct large (> 1000 kb) CNVs have been linked to rare disease phenotypes, and they may contribute to common polygenic diseases [2].

Numerous methods for the detection of CNVs have been established throughout the past decades. Initially, targeted gene panel methods such as quantitative polymerase chain reaction (qPCR) and multiplex ligation-dependent probe amplification (MLPA) were used. However, the introduction of the genome-wide approaches offered a significant advance in the CNV detection methods. Microarray-based methods such as array-CGH (comparative genomic hybridization) and single nucleotide polymorphism (SNP)-array allow the investigation of CNVs [3, 4], and more recently next-generation sequencing (NGS) [5] data are being used for CNV detection.

Despite the evolution of NGS-based methods, microarrays are still often the first tier solution for whole genome studies due to their comparatively lower cost and broad applicability. A large number of studies have investigated rare CNVs using microarray based genotyping data and yielded important insights [6–12]. These investigations typically involve intricate procedures, necessitating multiple analyses, careful choice of software, calibration of sensitivity to parameters and their thresholds, and execution setting. Computational and scientific outcomes therefore hinge upon automation and thorough documentation of implementation specifics. Standardized basic protocols for calling CNVs and performing association tests have been proposed by others, such as in Lin et al*.* [13], however a comprehensive simple-to-use bioinformatic implementation has not been provided.

Conducting a case–control study based on rare CNVs involves several critical steps: (1) CNV detection, (2) quality control, (3) burden analysis, and (4) gene-set enrichment analysis. High-throughput genomic technologies, commonly employed in genome-wide association studies (GWAS), provide the signal intensity data necessary for CNV detection. Subsequently, tools like PennCNV [14] and Plink [15] are typically used for the case–control analysis of CNV, focusing on individual-based CNV calls, and rare CNVs, respectively. Conducting such analyses therefore requires adeptly applying and coordinating multiple advanced bioinformatic software, but to the best of our knowledge a bioinformatic pipeline implementing rare CNV analysis in a structured, flexible, and scalable manner remains missing.

In this work, we present a generic bioinformatic solution for identifying rare CNVs in case–control studies. Our main goal is to provide a flexible tool that enables users to conduct rare CNV analysis using SNP array data from different case–control studies.

### Implementation

**Fig. 1:**
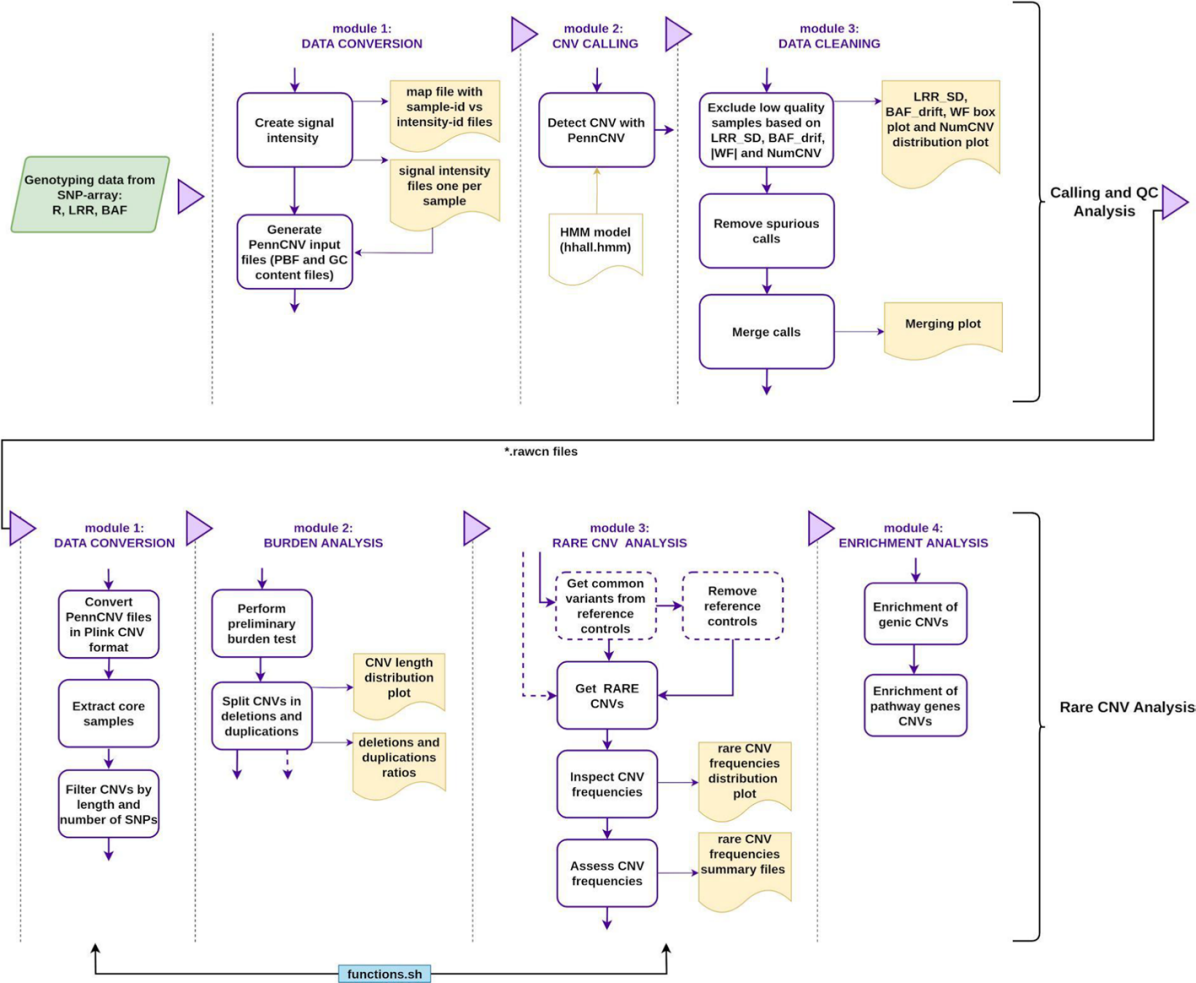
Rare CNVs workflow. The pipeline consists of two major sub-pipelines: (1) Calling and quality control (QC) analysis, which uses the SNP-array genotyping data (green box) as an input to retain good-quality samples and high-quality calls. (2) Rare CNV analysis, which takes samples and calls from the calling and QC sub-pipeline output, and after the data format conversion, performs the burden, rare CNV and enrichment analyses. Black dotted lines split each analysis in their corresponding modules, purple boxes represent a specific task in each module, yellow boxes show representative outputs (files and/or plots), yellow line box represents an external dependency, and the blue box represents external functions used by some modules. Dotted purple boxes are optional tasks which could be easily removed or changed to adapt the pipeline to the study requirements

**Fig. 2:**
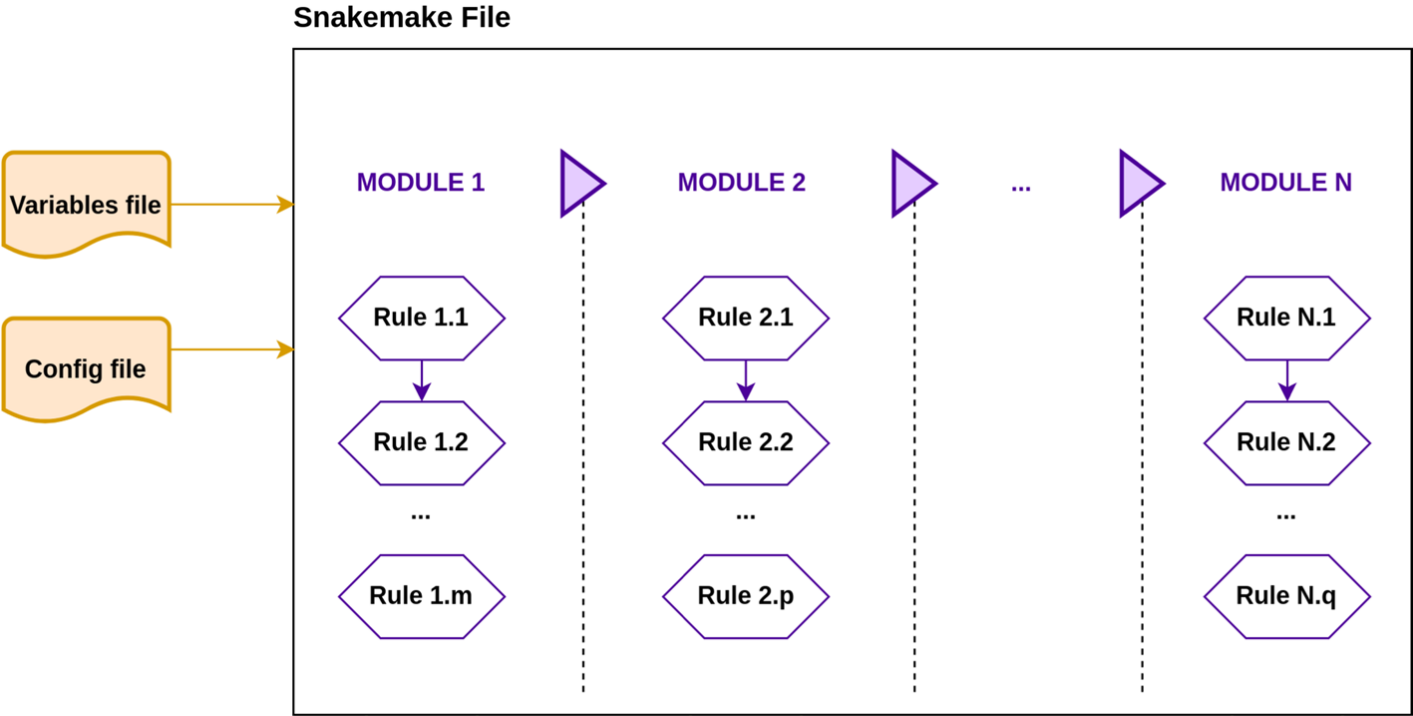
Pipeline structure based on snakemake modules. Both our sub-pipelines are organized in modules, each module containing one or more rules. Modules and rules can be modified, added or removed according to the analysis requirements. The list of modules should be included in the snakemake executable file and the description of variables, files and paths should be included in the variables and config files

**Fig. 3:**
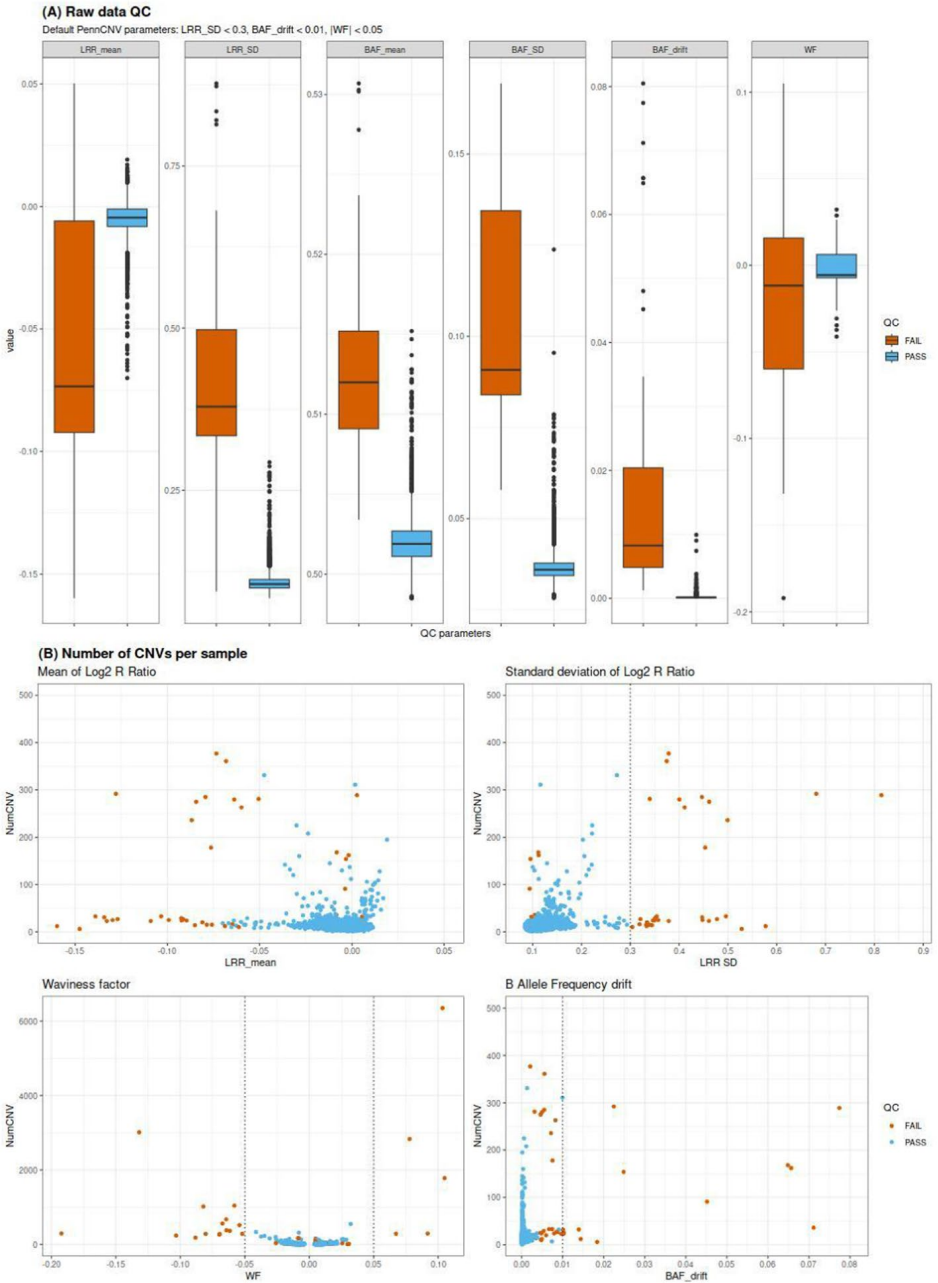
Quality control plots. **(A)** Sample quality parameters: Red boxes show samples which fail the inclusion criteria based on the PennCNV QC threshold (LRR_SD < 0.3 & BAF_drift < 0.01 & |WF|< 0.05). Blue boxes show samples which pass the quality control. **(B)** The distribution of the number of CNVs per sample. Samples with an excessive number of CNVs should be considered for exclusion because it can indicate low data quality. A threshold for the number of CNVs per sample (NumCNV) can be defined through visual inspection, considering its distribution around the exclusion criteria threshold values based on PennCNV statistics. In Addison's study (Artaza et al. [23]), samples with NumCNV > 50 were removed. Y-axis was truncated in 500 to improve the data visualization

**Fig. 4:**
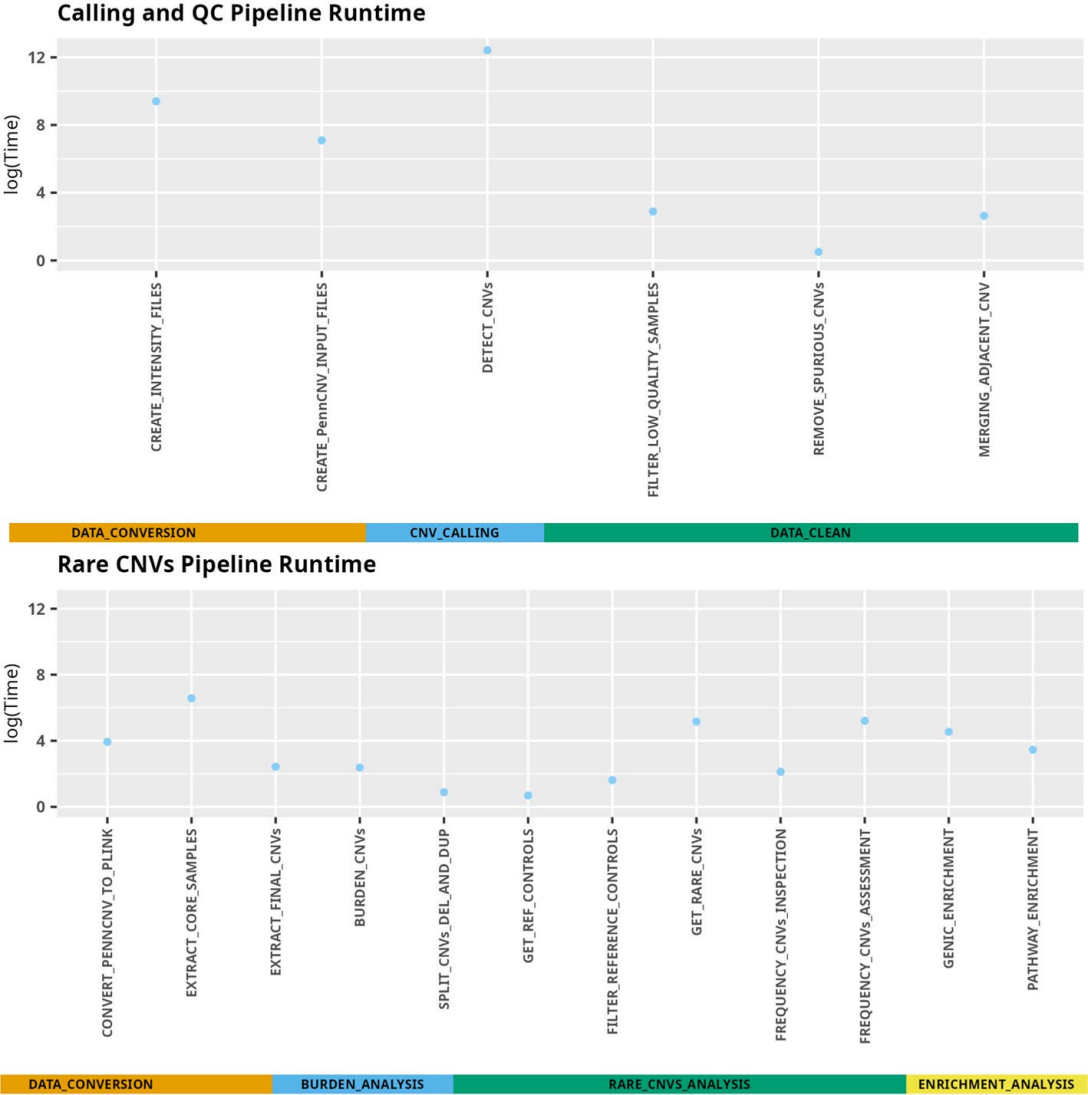
Pipeline performance. Calling and QC analysis sub-pipeline and rare-CNVs analysis sub-pipeline for 6,112 samples, 700,079 markers (genotyping data from Illumina GSA), and 98,702 calls detected. Time in seconds, in logarithmic scale, is plotted for each module-rule. Calling and QC analysis runtime was 72.31 h (260,320 s), and rare CNVs analysis runtime was 21.5 min (1290 s)

We have employed the Snakemake workflow [16] engine to construct a robust pipeline consisting of two sub-pipelines: (1) calling and QC analysis and (2) rare CNV analysis (Fig. 1). The code is modular and rule-based, using the modular configuration allowed by Snakemake (Fig. 2). Notably, if input files are missing for any rule, Snakemake will report it and the execution will be stopped. Files generated previously in successfully executed rules will be preserved. The next execution will start from the last rule completed. Moreover, if an execution error occurs, any corrupted output file is automatically deleted to maintain consistency. The rule-based structure enables automation while maintaining flexibility: Both sub-pipelines can be modified according to the nature of the study through parameters, software, or the addition of custom code. To illustrate this feature, instructions on how to adapt the input file format in the calling and QC sub-pipeline are described in the Pipeline Guide available in our RareCNVsAnalysis GitHub repository [17] under the section *Input Files Specification*. In addition, configuration files (such as variables.py and dependenciesenv.yml) are provided to facilitate the modification of the default value of parameters and the inclusion of new software (or a different version)(see Pipeline Guide Fig. 3 and Pipeline Guide Fig. 4 in the GitHub repository). Also, code that is executed in many rules can be added in the external functions.sh file to enhance the pipeline´s modularity, clarity and efficiency. Both sub-pipelines further generate execution logs, along with diagnostic plots produced using the R programming language [18]. Most of the dependencies are managed using Conda through the Snakemake Integrated Package Management [19]. Dependencies not available via Conda should be installed following the installation guide included in the GitHub repository. The pipeline is open source, released as a permissive MIT license [20], and the code is available along with documentation. Additionally, a Docker version of the pipeline is available in the GitHub repository alongside the main pipeline code. It allows running both sub-pipelines via Docker with full functionality. Detailed information regarding configuration files, input and output formats and contents for each module and rules are described in the pipelines guide available for download from RareCNVsAnalysis Github repository under manual/Rare_CNVs_pipeline_guide.pdf.

### Calling and quality control analysis

The first part of the pipeline consists of the calling and quality control analysis sub-pipeline (Fig. 1). This sub-pipeline executes a number of standard quality procedures and generates statistics and plots to guide the users when tuning the parameters to fit the study-specific needs and to ensure that the steps are performed as expected. It uses the SNP-array genotyping signal intensity values (Log R Ratio and B Allele Frequency) for all markers in all samples in text format (Pipeline Guide, Module Data Conversion). The cohort-wide signal intensity file is subsequently processed to generate an individual signal intensity file per sample which is utilized in the PennCNV calling process. Additionally, the population frequency of B allele (PFB) and the GCModel files are generated in this step since PennCNV relies on these for accurate CNV detection (more details in https://penncnv.openbioinformatics.org). After CNV detection, low quality samples are excluded based on standard genotyping quality metrics: LRR (Log R Ratio), BAF_drift (B Allele Frequency drift), WF (Waviness Factor), and NumCNVs (number of called CNVs). The sub-pipeline generates several plots that should be used by the user to inspect the performance of these quality metrics in samples meeting or failing the exclusion criteria and help guide the user to set their study-specific threshold values (Fig. 3). These thresholds, and the inclusion of other parameters (LRR_mean, LRR_median, LRR_SD, BAF_mean, BAF_median, BAF_SD) can be customized in the parameters file variables.py (Pipeline Guide, Pipeline Description). Calls detected in challenging genomic regions such as the Human leukocyte antigen (HLA), and the regions near the centromeres and the telomeres are considered spurious and are removed [21]. The genomic coordinates of these regions must be contained in external files which will be set into the configuration file config.json. Finally, the sub-pipeline merges adjacent CNV calls to mitigate the tendency of many CNV-callers to artificially split larger CNVs into smaller segments (Supplementary Fig. 1). This analysis generates a set of high-quality CNVs that will serve as the basis for further investigation of rare CNVs.

We have built the calling and QC sub-pipeline around the Illumina genotyping SNP array and formats, but it is possible to adapt it to support Affymetrix [22] data too. The user can do this by preparing input file formats according to the PennCNV requirements (see Pipeline Guide, Input Files Specification).

### Rare CNVs analysis

After the calling and QC analysis, the rare CNVs analysis can be performed using the samples and calls obtained in the previous section (Fig. 1 and Pipeline Guide, Rare CNV pipeline). These samples and calls in PennCNV format are first converted to Plink files. Only *core samples*, defined as unrelated and genetically unstratified, are retained, in order to avoid confounding effects [24, 25]. This task requires the users to provide a list with the identifiers of the core samples. These samples can be identified with a principal component analysis (PCA) or multidimensional scaling (MDS), while the genetic relatedness of the individuals can be based on identity by descent (IBD) analysis (e.g. $\widehat{\pi }\le 0.1$). Small CNV calls are usually not reliably detectable by SNP arrays [26, 27], therefore only CNVs larger than 50 kb and covered by more than 5 probes are retained at this stage. Default values can be modified in the parameters file (Pipeline Guide, Pipeline Description).

After sample filtering and exclusion of CNV by size, a global burden test in cases versus controls is conducted using Plink software. The burden test is performed for four key metrics: (1) number of segments (RATE), (2) proportion of samples with one or more segments (PROP), (3) total kb length spanned (TOTKB), and (4) average segment size (AVGKB). Subsequently, CNVs are divided into deletions and duplications and pooled by length to calculate the CNV frequency in cases versus controls and the CNV distribution within specific length intervals (Supplementary Fig.2). By default, the rare CNV sub-pipeline defines CNV size thresholds intervals as 50 kb, 100 kb, 200 kb, 500 kb, and 1,000 kb. Users can customize these thresholds in the parameters file (Pipeline Guide, Pipeline Description).

Following the rare CNVs analysis, the sub-pipeline proceeds to extract rare deletions and duplications. This involves identifying common CNVs with frequencies greater than or equal to a user-defined threshold from a subset of healthy control individuals in the study cohort. To calculate the CNV frequency, the Plink overlapping strategy is used. It assigns a specific count to each CNV that represents the number of CNVs (including itself) that overlap with at least 50% of its region. The CNV overlap definition is based on a union intersection approach (Supplementary Fig. 3). The subset of healthy individuals involved in the common CNVs identification, are subsequently excluded from further analysis to avoid bias to the test statistics. Using the common CNVs as reference, common variants are filtered out from both the cases and remaining control samples by removing all CNVs with at least 50% overlap with common CNVs. This task is carried out using the BEDTools suite [28]. Frequency histograms are generated for quality control of the procedure (Supplementary Fig. 4 and 5). It is important to note that our suggested approach to identify rare CNVs can be adapted or modified according to the study strategy. Following this, differences in the frequencies among cases and controls are first assessed for all deletions and duplications, and then, the differences are evaluated for intervals of binned CNV sizes. Summary statistics are generated containing the frequencies for common and rare CNVs in different interval sizes, along with two proportion test statistics and odds ratios (OR) estimation using R version 3.6.3 [18], specifically the *stats* and *fmsb* packages. These results are represented graphically as forest plots, with the confidence intervals of frequencies within each CNVs interval size, alongside the associated p-value (Supplementary Fig. 6 and 7).

In the final stage of the rare CNV analysis sub-pipeline, the Plink gene set enrichment method is employed. This test compares the rate of CNVs impacting specific gene sets in cases versus controls, while taking into account gene size and differences in CNV rate [29]. The sub-pipeline includes two tests by default: the enrichment of genic CNVs (asking the question whether there is a general enrichment of genes among case CNVs), and the enrichment of pathway (or a predefined list of) genes, relative to all CNVs (determining whether there is a subset of genes enriched, relative to the whole genome). Both tests are based on a permutation test with N = 10,000 null permutations to generate empirical p-values (N can be modified inside the enrichment analysis module). The genomic coordinates of the genes, as well as the pathways to be tested are provided as configuration files to the sub-pipeline. The enrichment test performs a generalized linear model-based (GLM-based) CNV burden test, and evaluates gene counts (GCNT), number of segments or CNVs (NSEG), and average size of CNVs (AVGKB) using logistic regression.

### External code and logs

A rule in a specific module can include inline code in Python or shell commands. However, extensive code within a single rule might hinder the module-rule modification. An external file (function.sh) containing shell functions used by some modules (Fig. 1) is included with the pipeline utilities, making the inclusion or modification of external shell code clearer and simpler.

Both the calling and QC sub-pipeline and rare CNVs sub-pipeline automatically generate the log text files (inside the logs directory) with relevant information for each module, such as number of samples included/excluded, number of calls filtered, burden summary and enrichment summary. Logs can be used to create a report including overall information as presented in Table 1 and Supplementary Table 2 and Supplementary Table 3.

**Table 1:** Calling and QC sub-pipeline report

Module 1: Data Conversion						
Generate signal intensity file	**700,079** markers **6,112** samples					

Module 2: Data Calling						
	Initial samples	Final samples	Lost	Initial calls	Final calls	Lost
Raw data	6,112	–	–	**98,702**	–	–
Module 3: Data Clean						
Filters	Initial samples	Final samples	Lost	Initial calls	Final calls	Lost
Default parameters*	6,112	6,012	100	98,702	71,010	27,692
Clean Immunoglobulin regions	6,012	6,012	–	71,010	70,436	574
Clean centromere and telomere regions	6,012	6,012	–	70,436	63,202	7,234
Merging calls**	6,012	**6,012**	–	63,202	**60,705**	2,497

Module 3: Data Clean						
Filters	Initial samples	Final samples	Lost	Initial calls	Final calls	Lost
Default parameters*	6,112	6,012	100	98,702	71,010	27,692
Clean Immunoglobulin regions	6,012	6,012	–	71,010	70,436	574
Clean centromere and telomere regions	6,012	6,012	–	70,436	63,202	7,234
Merging calls**	6,012	**6,012**	–	63,202	**60,705**	2,497

The table summarizes the samples included and excluded at each module in the calling and QC sub–pipeline. Final samples and calls, after QC, are in bold

^*^LRR_SD < 0.3, BAF_drift < 0.01, |WF|< 0.05, NumCNV > 50

^**^fraction: 0.5 and 0.4. In this step, calls were not lost, but the number decreased because two or more calls can be combined into a unique

## Performance

This pipeline executes non-parallel tasks, although Snakemake can automatically determine which parts of the workflow can be run in parallel, decreasing the execution time of some modules. Figure 4 shows the runtime for both the calling and QC sub-pipeline and rare CNV sub-pipeline, for genotyping data (from Illumina GSA) of 6,112 samples, 700,079 markers, and 98,702 calls detected. The calling and QC sub-pipeline execution time, approximately 72 h, or approximately 0.71 min per sample, takes most of the total time of the execution, especially modules which perform the data conversion from the signal intensity values to PennCNV, and CNV calling. The per sample time usage was similar (0.73 min/sample) when running it on half the cohort. It should be mentioned that these modules will be executed only on the first run. The downstream rules, directly involved with samples and calls quality, can be modified and the calling and QC sub-pipeline can be executed again, skipping the run of the previous modules which decreases the execution time substantially. A similar approach is applied for the rare CNVs sub-pipeline.

Due to the security requirements for personally identifiable data used in this performance testing, we used the TRE provided by the HUNT cloud secure solutions for scientific cloud computing (ntnu.edu/mh/huntcloud):

Operative system: Ubuntu 18.04.6 LTS (GNU/Linux 4.15.0–210-generic x86_64).

Architecture: x86_64.

CPU op-mode(s): 32-bit, 64-bit.

CPU(s): 32.

Model name: Intel Core Processor (Broadwell, no TSX, IBRS).

CPU MHz: 2095.074.

Memory: 64 GB.

Total runtime: 72.67 h.

Moreover, this pipeline can be run on a standard desktop computer. A basic test was performed using a small demo data (12 samples, 654,028 markers and 472 calls detected) downloaded from Illumina in an Ubuntu virtual machine (see Pipeline Execution in RareCNVsAnalysis Github project):

Operative system: Ubuntu 22.04.4 LTS.

Architecture: x86_64.

CPU op-mode(s): 32-bit, 64-bit.

Model name: 11th Gen Intel(R) Core(TM) i7-1165G7 @ 2.80 GHz.

Memory: 4 GB.

Total runtime: 6.21 min.

## Results and discussion

We have created a versatile pipeline for detection and analysis of CNVs from SNP arrays. To demonstrate the use of the pipeline we applied it to a case–control study in Addison’s disease where the results are presented in more detail in Artaza et. al [23]. Samples were genotyped with Illumina Infinium Global Screening Array 1.0. CNVs were called and quality controlled using the calling and QC sub-pipeline. The box plots displaying PennCNV statistics values (LRR_mean, LRR_SD, BAF_mean, BAF_SD, BAF_drift and |WF|) were generated to assess the quality of the samples (Fig. 3A). This plot illustrates samples meeting or failing the exclusion criteria based on the PennCNV QC threshold. Reduced overlap in the side-by-side box plots signifies a robust quality predictor (LRR_SD in this study). Furthermore, an abnormally high count of CNVs in a sample (NumCNV) suggests a low quality at a sample-level; such samples should be therefore excluded. The NumCNV threshold (> 50 in this study) can be established by inspecting the correspondence among samples failing or passing QC and the NumCNV (Fig. 3B). After sample QC, potentially artificial CNV calls were removed from repeat-rich genomic regions such as HLA, telomeric, and centromeric regions, and then CNVs were merged to produce a set of high-quality CNV calls (Supplementary Fig. 8). Table 1 illustrates the main steps of the calling and QC sub-pipeline and the number of samples and CNVs included and excluded in each step.

After filtering samples and CNVs, the sub-pipeline for the analysis of rare CNVs was executed. First, the PennCNV sample files were converted to Plink format and then, only unrelated ($\widehat{\pi }\le 0.1$) individuals of European descent were retained. CNVs above 50 kb in length and spanning more than five markers were selected (default values can be changed in the sub-pipeline parameter file) and a burden test for all CNVs was performed, which showed no significant differences in cases compared to controls in the four metrics, RATE (Number of segments), PROP (Proportion of samples with one or more segment), TOTKB (Total kb length spanned) and AVGKB (Average segment size) (Supplementary Fig. 9). Continuing with the burden analysis, CNVs were classified into deletions and duplications, binned by length (by default 50 kb, 100 kb, 200, 500 kb and > 1,000 kb) and further the ratios in controls and cases were calculated (Supplementary Table 1. Once the burden analysis was finalized, the sub-pipeline proceeded to rare CNV analysis, in which the rare deletions and duplications were extracted and evaluated for differences in frequency between cases and controls. For this study in particular, a subset of controls (200 individuals) previously selected were used as a reference to identify the common variants. Variants with count ≥ 4 (i.e. ≥ 2% carrier frequency) were classified as common variants. Subsequently, any CNVs overlapping at least 50% of length with these common variants were excluded to retain the rare variants with a frequency below 1% (carrier frequency < 2%). The carrier frequency plot distribution for rare deletions and duplications, generated by the sub-pipeline, enabled us to inspect these frequencies. The obtained frequencies fell within the predefined threshold for this study (Supplementary Fig. 4 and 5). Next, the sub-pipeline evaluated the cumulative distribution of CNV frequencies across five interval sizes (50–100, 100–200, 200–500, 500–1,000 kb and > 1,000 kb), calculating a two proportion test statistic and odds ratios (ORs). The results were then compiled in a summary file, alongside the forest plots (Table 2 and Table 3, and Supplementary Fig. 6 and 7). The analysis which is described in detail in Artaza et al*.* [23] uncovered a higher frequency for the largest rare deletions (> 1,000 kb) among cases (n = 13/1182) compared to controls (n = 10/3810) (OR = 4.23, 95% CI 1.85–9.66, *p* = 0.0002). Finally, the sub-pipeline performed the case–control gene-set enrichment test for two candidate gene-set lists, primary immunodeficiency and congenital adrenal hypoplasia panels from the Genomics England PanelApp [30]. Based on the test results, no evidence supporting an overall enrichment of rare CNVs overlapping with immune related genes was observed [23] (Supplementary Fig. 10).

**Table 2:** Overall rare deletions and duplications frequency distribution

CNV	Cases	Controls	Cases_freq	Controls_freq	P.value	OR	X95.CI	P
DELs	827	2615	0.6997	0.6864	0.4077	1.0646	0.9236,1.2269	0.3876
DUPs	721	2367	0.6100	0.6213	0.5073	0.9535	0.8339,1.0901	0.4857

The table shows data directly extracted from a summary text file. The table format can be adjusted by the user. **CNV**: CNV type, **Cases/Controls**: number of CNVs (deletions or duplications) in each cohort. **Cases_freq/Controls_freq**: CNVs frequencies, ***P*.value**: two proportion test p-value, **OR**: odds ratio, **X95.C:** confidence interval at 95%, **P**: odds ratio p-value associate

**Table 3:** Rare CNV frequency distribution binning by size in cases vs. controls

Deletions								
Length	Cases	Controls	Cases_freq	Controls_freq	P.value	OR	X95.CI	P
50KB_100KB	435	1298	0.3680	0.3407	0.0911	1.1270	0.9837,1.2909	0.0846
100KB_200KB	260	919	0.2200	0.2412	0.1435	0.8871	0.7586,1.0372	0.1331
200KB_500KB	102	323	0.0863	0.0848	0.9174	1.0196	0.8078,1.2869	0.8703
500KB_1000KB	17	65	0.0144	0.0171	0.6158	0.8407	0.4909,1.4397	0.5269
1000KB_1000000KB	13	10	0.0110	0.0026	0.0005	4.2258	1.8481,9.6622	0.0002

Duplications								
Length	Cases	Controls	Cases_freq	Controls_freq	P.value	OR	X95.CI	P
50KB_100KB	297	1050	0.2513	0.2756	0.1078	0.8821	0.7597,1.0242	0.0998
100KB_200KB	204	614	0.1726	0.1612	0.3773	1.0857	0.9125,1.2918	0.3536
200KB_500KB	157	488	0.1328	0.1281	0.7077	1.0427	0.8596,1.2646	0.6712
500KB_1000KB	48	150	0.0406	0.0394	0.9161	1.0328	0.7411,1.4391	0.8488
1000KB_1000000KB	15	65	0.0127	0.0171	0.3614	0.7406	0.4207,1.3033	0.2960

The table shows data directly extracted from a summary text file. The table format can be adjusted by the user. **Length**: CNV length interval, **Cases/Controls**: number of CNVs (deletions or duplications) in each cohort. **Cases_freq/Controls_freq**: CNVs frequencies, **P.value**: two proportion test p-value, **OR**: odds ratio, **X95.C:** confidence interval at 95%, **P**: odds ratio p-value associate

## Conclusion

We present an automated, flexible, and scalable bioinformatic pipeline tailored for rare CNV analysis in case–control studies. Array technology has undergone a tremendous growth in both quantity and content over recent years. Although genotyping data facilitate CNV analysis, the major challenges in the CNV analysis involve the management of large volumes of data, advanced bioinformatics, and complex data interpretation. Addressing this, a pipeline that streamlines analyses, systematizing tasks, while maintaining flexibility is indispensable. Our pipeline provides the fundamental steps for rare CNVs analysis, enabling automation of analyses while maintaining flexibility. Beyond the analysis of rare CNVs, the design principles using standardized modules render the pipeline reusable across a broad spectrum of bioinformatic analyses .

## Supplementary Information

Supplementary file1 (PDF 306 KB)

## Data Availability

We applied the pipeline to a case-control study in Addison’s disease. This dataset is not publicly accessible due to privacy and consent concerns for research participants. Requests to access the datasets should be directed to ASBW (Anette.boe@uib.no). A demo data is available at GitHub (https://github.com/haydeeartaza/RareCNVsAnalysis. Project name: Rare CNVs Analysis Pipeline. Project home page: https://github.com/haydeeartaza/RareCNVsAnalysis. Operating system(s): Linux, MacOS. Programming language: R, Shell Scripting, Python. License: MIT license. Any restrictions to use by non-academics: none. We used our in-house data to test the pipeline due to paucity of suitable publicly available datasets.

## Abbreviations

CNV: Copy number variation
SNP: Single nucleotide polymorphism
GWAS: Genome wide association study
LRR: Log R Ratio
BAF: B allele frequency
WF: Waviness Factor
NumCNVs: Number of called CNVs
PFB: Population frequency of B allele
PCA: Principal component analysis
MDS: Multidimensional scaling
IBD: Identity by descent
