# Supplementary material for: Rare copy number variant analysis in case–control studies using snp array data: a scalable and automated data analysis pipeline
Source: BMC Bioinformatics. 2024 Nov 15;25:357. doi: 10.1186/s12859-024-05979-0 (PMC11566566; doi:10.1186/s12859-024-05979-0)
Supplement: Supplementary file 1 — Supplementary file1 (PDF 306 KB) [file 12859_2024_5979_MOESM1_ESM.pdf]

Supplementary Figures and Tables

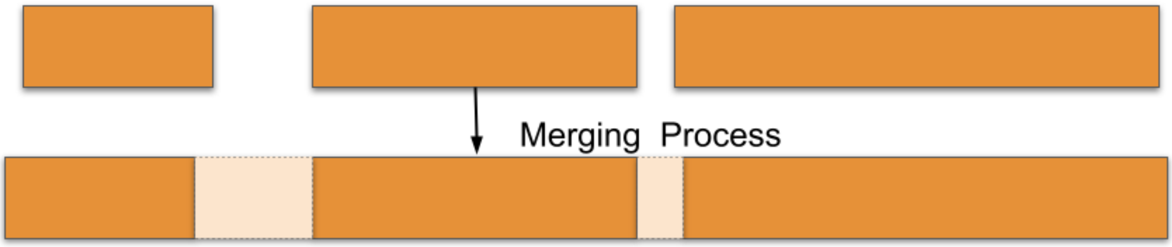

Supplementary Figure 1. Merging CNV calls. PennCNV has a tendency to divide large CNVs into smaller fragments. The pipeline combines neighboring CNVs into segments, with the default setting merging adjacent calls when the fraction of the gap length between two calls compared to the total length of the resulting merged call is <20%.

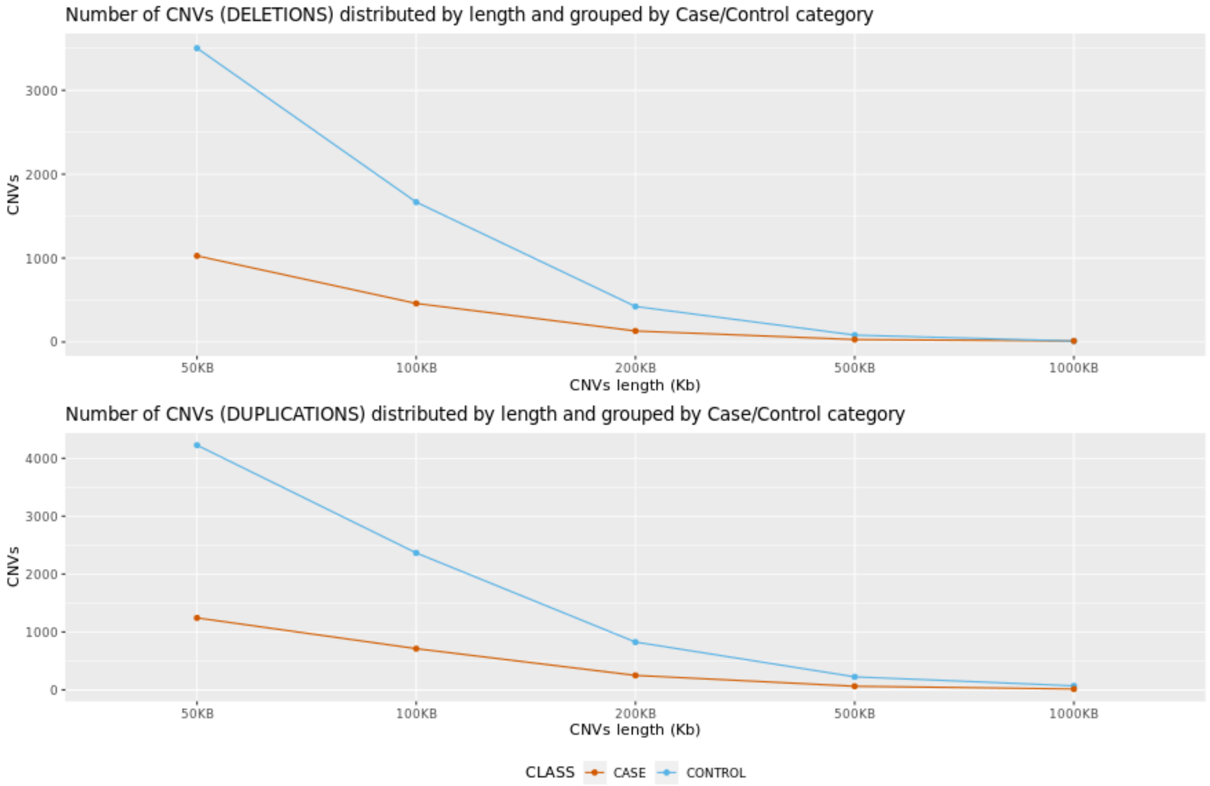

Supplementary Figure 2. Cumulative number of CNVs by size. A good sanity check early on in the study can prevent inaccurate results and save time spent running the analysis. This plot confirms the natural accumulation tendency of CNVs according to their length and the uniform CNVs distribution in cases and controls (Artaza *et al*. doi:10.3389/fimmu.2024.1374499).

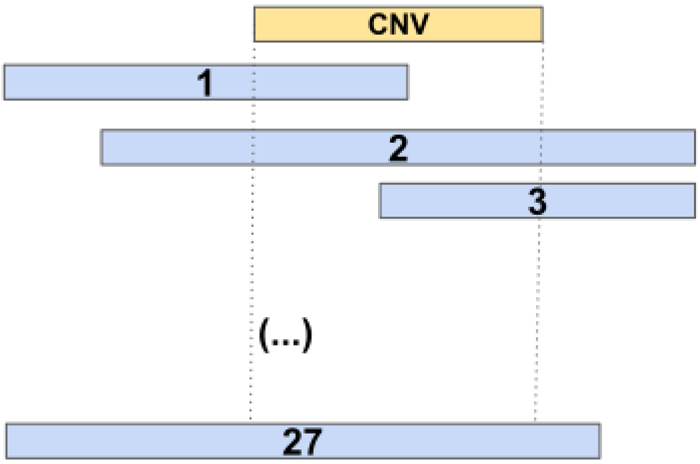

Supplementary Figure 3. CNV frequency calculation. For each CNV, the pipeline calculates the total number of CNVs that overlap with this CNV, including itself. The frequency is subsequently determined based on these counts. A 50% union overlap criterion is employed to ensure symmetry (i.e. if A overlaps B, then B must overlap A). In this example, the count for the target CNV region (in yellow) will be 28, indicating that 28 individuals harbor approximately the same CNV (in yellow) The frequency for this CNV is then calculated to be 28 divided by the total number of samples.

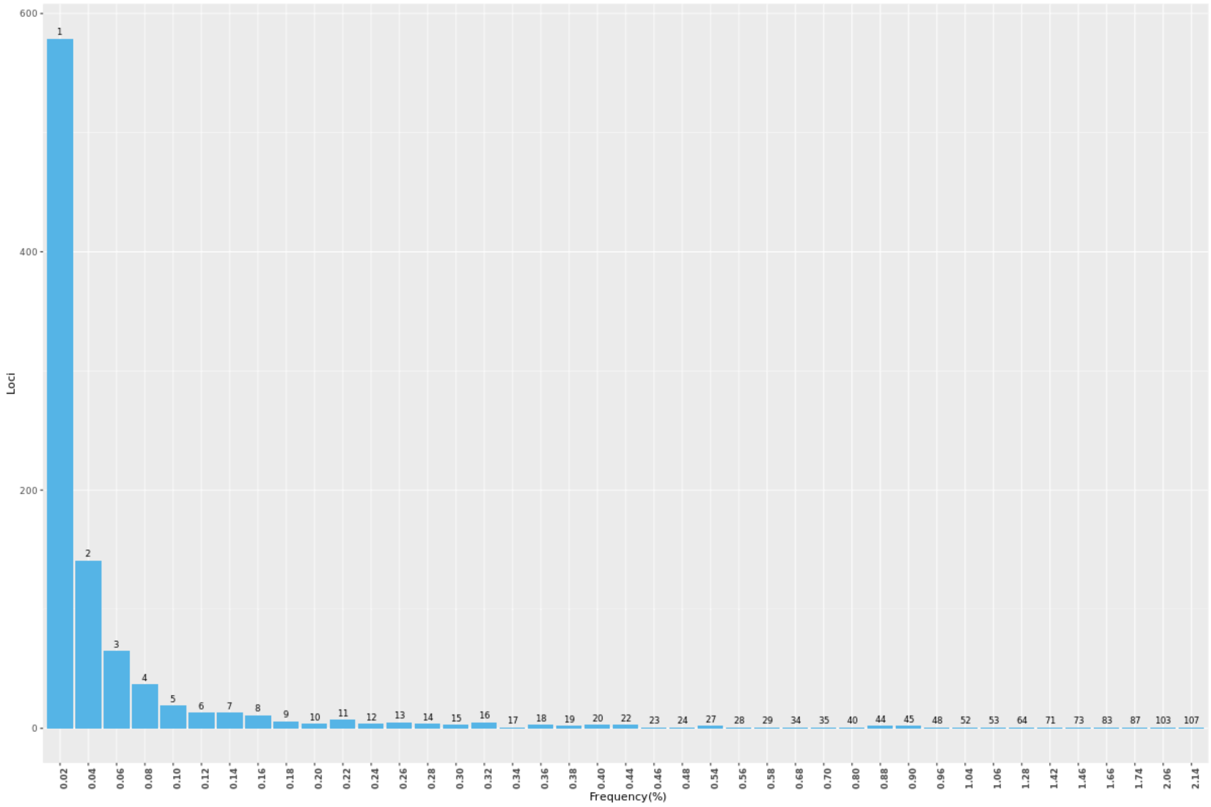

Supplementary Figure 4. Rare deletions frequency distribution. The Y axis shows the number of loci (regions), the X axis shows the frequency, and the number on top of the bar shows the corresponding number of deletions this frequency bin represents. E.g. 141 loci or regions (second bar size) contain 2 deletions (number in the bar) corresponding to a CNV frequency of ~0.04% (Artaza *et al.* doi:10.3389/fimmu.2024.1374499).

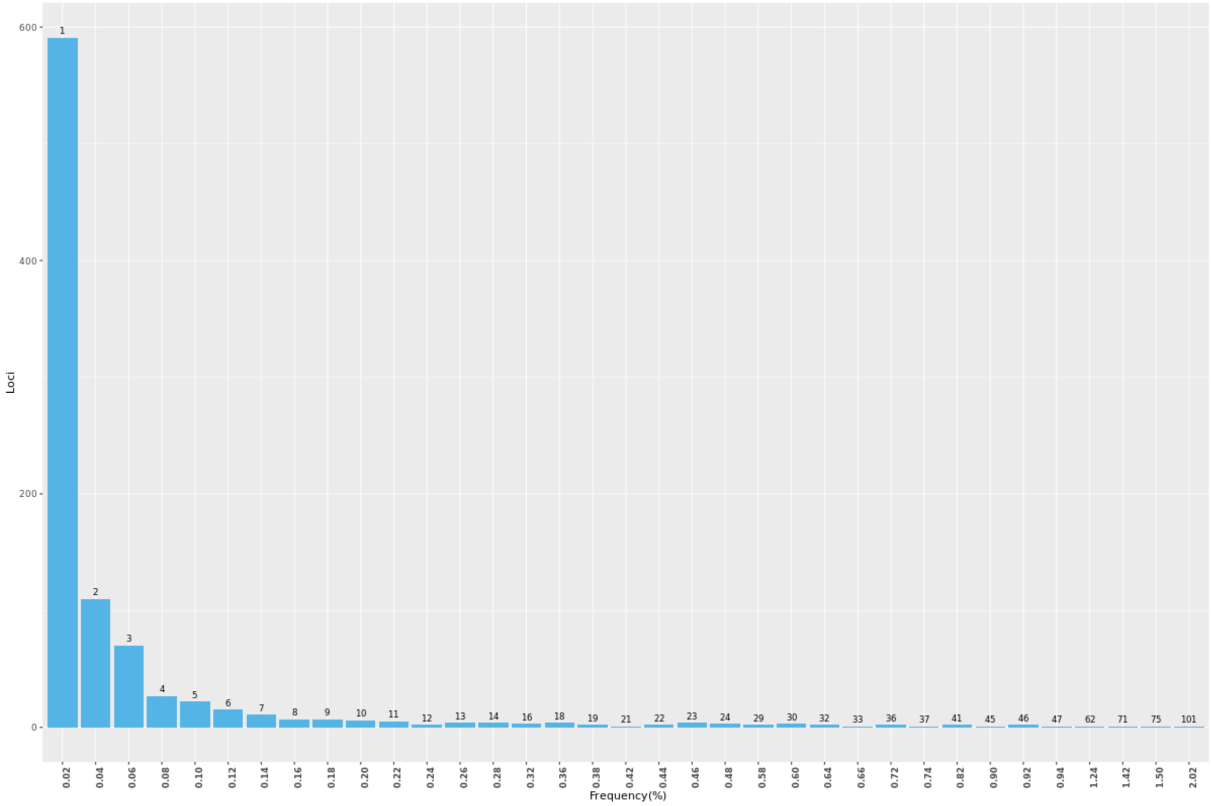

Supplementary Figure 5. Rare duplication frequency distribution. The Y axis shows the number of loci (regions), the X axis shows the frequency, and the number on top of the bar shows the corresponding number of deletions this frequency bin represents. E.g. ~600 loci or regions (first bar size) contain 1 duplication (number in the bar) corresponding to a CNV frequency of ~0.02% (Artaza *et al*. doi:10.3389/fimmu.2024.1374499).

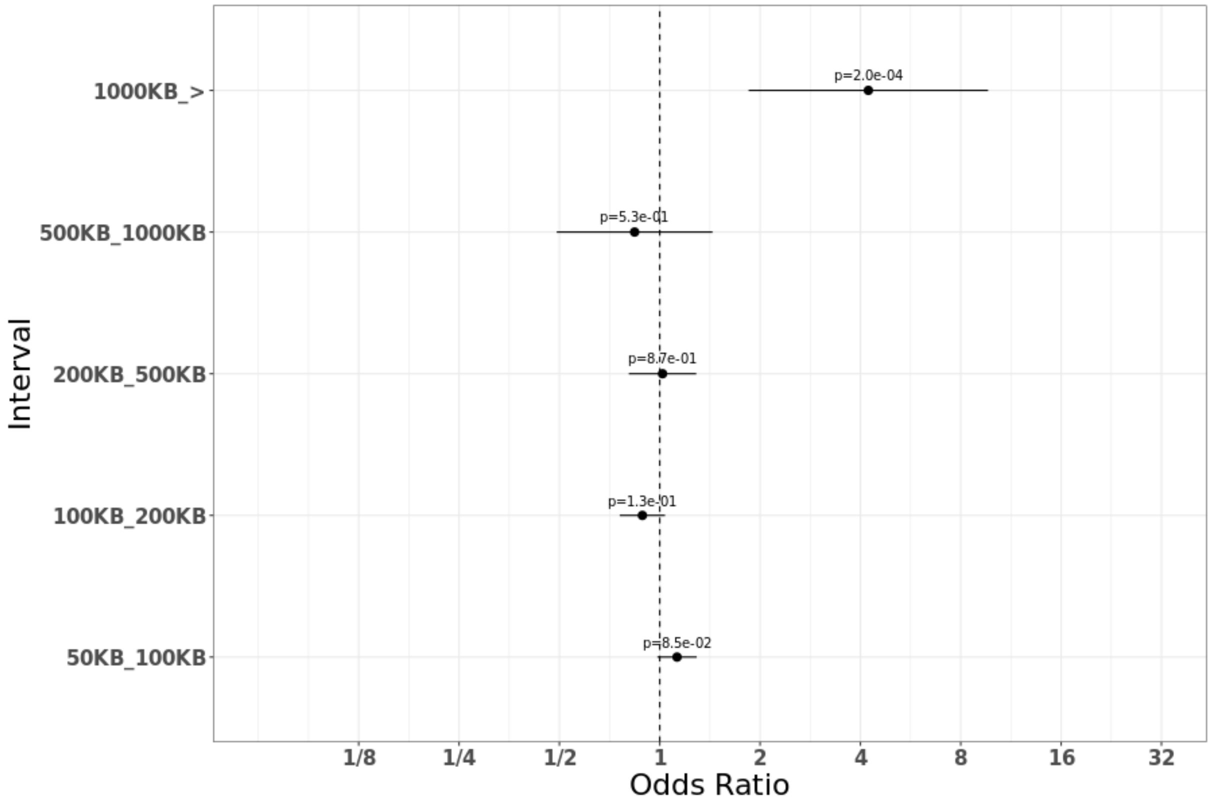

Supplementary Figure 6. Odds ratio for rare deletions distributed by size. Odds ratios shown by closed circles and whiskers represent the 95% confidence interval. The test P-value is located above the line. For long deletions (> 1000 kb) a significant difference in the frequency was found between cases and controls (Artaza *et al*. doi:10.3389/fimmu.2024.1374499).

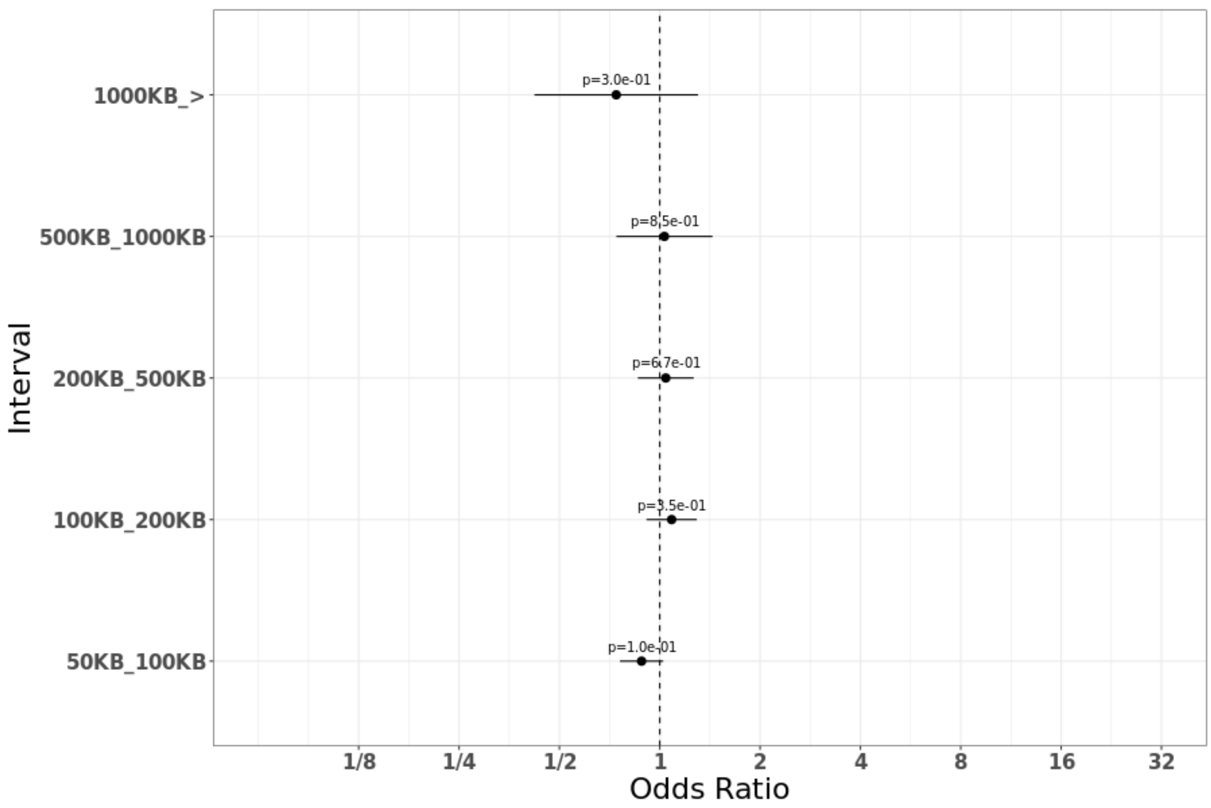

Supplementary Figure 7. Odds ratios for rare duplications distributed by size. Odds ratios are shown by closed circles and whiskers representing the 95% confidence interval. The P-value associated is located above the line. No significant difference in the frequencies were detected (Artaza *et al.* doi:10.3389/fimmu.2024.1374499).

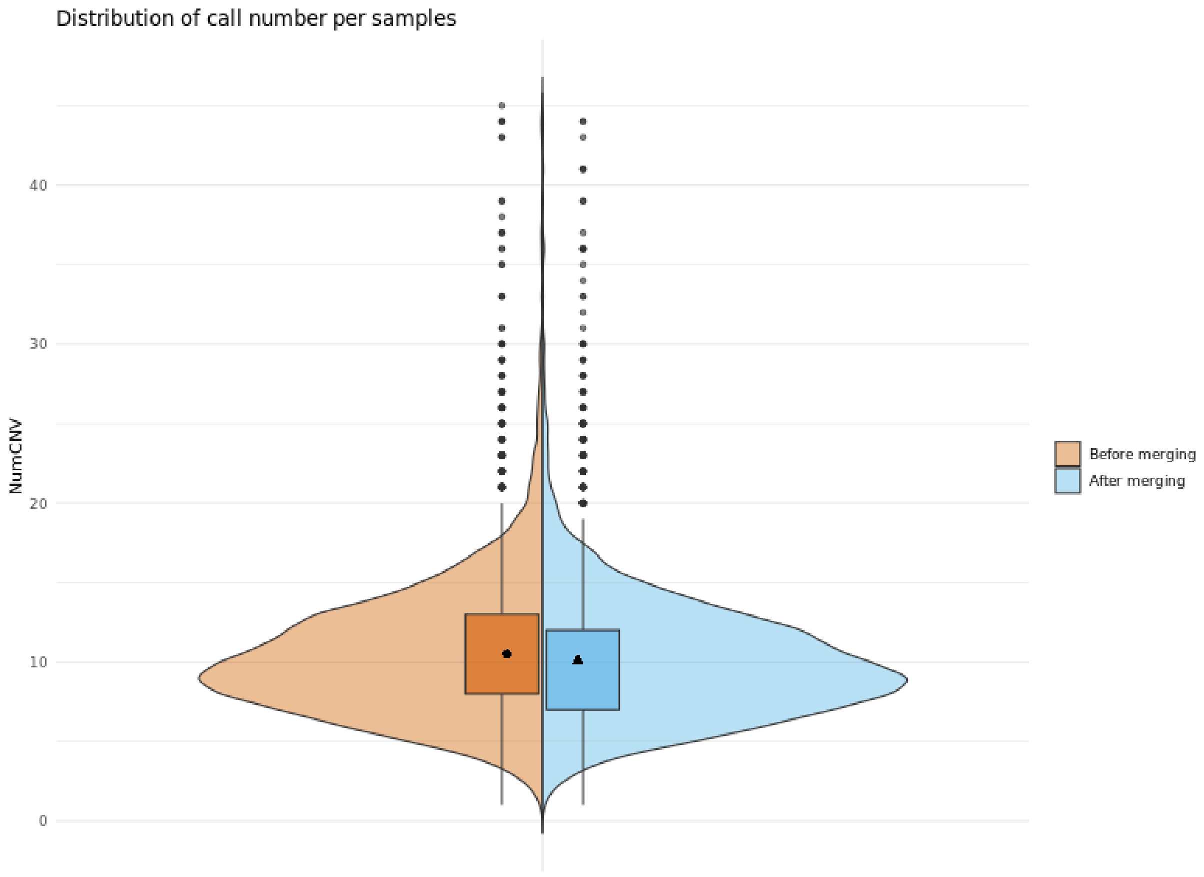

Supplementary Figure 8. The distribution of the number of calls per sample before and after the merging process. Following this process the number of calls per sample decreases as adjacent calls are merged into single calls (Artaza *et al.* doi:10.3389/fimmu.2024.1374499)..

(a) study_cnv.summary.mperm file

CHR	SNP	EMP1
S	RATE	0.567443
S	PROP	0.715528
S	KBTOT	0.339566
S	KBAVG	0.284772
S	GRATE	1
S	GPROP	1
S	GRICH	1

(b) study_cnv.grp.summary file

TEST	GRP	AFF	UNAFF
N	ALL	2270	7728
RATE	ALL	1.92	1.927
PROP	ALL	0.8477	0.8536
TOTKB	ALL	362.6	356.8
AVGKB	ALL	160.5	157.2

Supplementary Figure 9. Basic burden analysis outcome**.** The figure shows two files generated in the basic burden analysis for the four metrics RATE (Number of segments), PROP (Proportion of sample with one or more segment), TOTKB (Total kb length spanned), AVGKB (Average segment size): (a) permuted results for segment test, includes the standard empirical p-value; and (b) group cases (affected, AFF) and controls (unaffected, UNAFF) summary statistics (Artaza *et al.* doi:10.3389/fimmu.2024.1374499).

TEST	BETA	P
GCNT	0.0146582	0.400695
NSEG	-0.0204375	0.786693
AVGKB	0.000311012	0.320859

Supplementary Figure 10. Enrichment test analysis outcome. The enrichment test consists of a GLM-based CNV burden analysis, yielding logistic regression parameters associated with gene counts (GCNT), number of segments or CNVs (NSEG), the average size of CNVs (AVGKB), and their respective P-values (Artaza *et al.* doi:10.3389/fimmu.2024.1374499).

Supplementary Table 1. Burden analysis.

**Deletions**			
	length	num_CNVs	ratio
Controls	50KB	3502	0.87
	100KB	1667	0.42
	200KB	424	0.11
	500KB	83	0.02
	1000KB	12	0.00
Cases	50KB	1027	0.87
	100KB	460	0.39
	200KB	132	0.11
	500KB	30	0.03
	1000KB	13	0.01
**Duplications**			
	length	num_CNVs	ratio
Controls	50KB	4226	1.05
	100KB	2365	0.59
	200KB	826	0.21
	500KB	225	0.06
	1000KB	70	0.02
Cases	50KB	1243	1.05
	100KB	712	0.60
	200KB	250	0.21
	500KB	63	0.05
	1000KB	15	0.01

CNVs (deletions and duplications), distributed by length in controls and cases. [**num_CNVs]**: total number of CNVs in each interval length, [**ratio]**: number of CNVs divided by number of cases or controls respectively (Artaza *et al.* doi:10.3389/fimmu.2024.1374499).

Supplementary Table 2. Sample and call numbers in the rare CNV pipeline.

	Total samples	Cases	Controls	Males	Females	CNV calls
Raw data	6112*	1526	4471	3155	2946	98702
High quality **core** samples	5192	1182	4010	2705	2487	52144
Samples with CNVs > 50KB & 5 SNPs	**4425**	1002	3423	2302	2123	**9998**

Includes information on the cohort, gender and CNV calls. These details are documented in the log file of the data conversion module (Artaza *et al.* doi:10.3389/fimmu.2024.1374499).

(*) 7 samples without classification: case/control

Supplementary Table 3. Rare CNVs extraction strategy.

Type	Step		CNVs	Samples
All	1.	Initial core data (high quality samples and CNVs > 50KB and covered by at least 5 SNPs)	9998	4425
	2.	Define 200 reference controls	442	200
	3.	Remove 200 reference controls defined in (2) from initial data	**9556**	**4225**
DELETIONS	4.	Initial deletions in core data (high quality samples and CNVs > 50KB covered by at least 5 SNPs)	4351	2903
	5.	Common deletions (frequency >= 2%) from 200 reference controls	46	50
	6.	Identify CNVs in (4) overlapping at least 50% with common variants (reciprocal)	909	856
	7.	Remove common deletions from the dataset in (6)	**3442**	2470
DUPLICATIONS	8.	Initial duplications in core data (high quality samples and CNVs > 50KB and covered by at least 5 SNPs)	5205	3167
	9.	Common duplications (frequency >= 2%) from 200 reference controls	119	99
	10.	Identify CNVs in (8) overlapping at least 50% with common variants (reciprocal)	2117	1746
	11.	Remove common duplications from the dataset in (10)	**3088**	2212

Following QC and filtering CNVs > 50 kb and covered by more than five probes, 200 reference control individuals (specific thresholds for this example) are extracted from the control cohort to identify common variants (frequency >= 2%). These individuals are then removed from the control group, along with their respective CNV calls. After that, rare deletions and duplications are extracted, with CNVs overlapping at least 50% of the reference common variants being excluded. These details are documented in the log file of the rare CNV analysis module (Artaza *et al.* doi:10.3389/fimmu.2024.1374499).
